# Cytomegalovirus-based vaccine expressing Ebola virus glycoprotein protects nonhuman primates from Ebola virus infection

**DOI:** 10.1038/srep21674

**Published:** 2016-02-15

**Authors:** Andrea Marzi, Aisling A. Murphy, Friederike Feldmann, Christopher J. Parkins, Elaine Haddock, Patrick W. Hanley, Matthew J. Emery, Flora Engelmann, Ilhem Messaoudi, Heinz Feldmann, Michael A. Jarvis

**Affiliations:** 1 Laboratory of Virology, Division of Intramural Research, National Institute of Allergy and Infectious Diseases, National Institutes of Health, Hamilton, Montana, United States of America; 2 School of Biomedical and Healthcare Sciences, University of Plymouth, Devon, United Kingdom; 3 Rocky Mountain Veterinary Branch, National Institute of Allergy and Infectious Diseases, National Institutes of Health, Hamilton, Montana, United States of America; 4 Vaccine and Gene Therapy Institute, Oregon Health & Sciences University, Portland, Oregon, United States of America; 5 School of Biological Sciences, University of Plymouth, Devon, United Kingdom; 6 School of Medicine, University of California, Riverside, California, United States of America

## Abstract

Ebolaviruses pose significant public health problems due to their high lethality, unpredictable emergence, and localization to the poorest areas of the world. In addition to implementation of standard public health control procedures, a number of experimental human vaccines are being explored as a further means for outbreak control. Recombinant cytomegalovirus (CMV)-based vectors are a novel vaccine platform that have been shown to induce substantial levels of durable, but primarily T-cell-biased responses against the encoded heterologous target antigen. Herein, we demonstrate the ability of rhesus CMV (RhCMV) expressing Ebola virus (EBOV) glycoprotein (GP) to provide protective immunity to rhesus macaques against lethal EBOV challenge. Surprisingly, vaccination was associated with high levels of GP-specific antibodies, but with no detectable GP-directed cellular immunity.

Ebola virus (EBOV), a member of the family *Filoviridae*, is a negative, nonsegmented, single-stranded RNA virus responsible for zoonotic emergence of hemorrhagic fever outbreaks in humans and nonhuman primates (NHPs) in regions of West and Central Africa^1^. A recent ecological niche mapping study based on all recorded zoonotic EBOV transmissions from bats and NHPs to humans between the first EBOV outbreak in 1976 through to 2014 has identified an area at risk for EBOV zoonoses representing 22 African countries and a population of 22 million^2^. In December 2013, an unprecedented EBOV outbreak starting in the West African country of Guinea spread to neighboring countries of Sierra Leone and Liberia resulting in the world’s largest Ebola hemorrhagic fever (EHF; recently also designated Ebola virus disease) epidemic^3^. EBOV is also highly lethal in great apes, and is regarded as a major threat to the survival of chimpanzees and gorillas in the wild^4^,^5^.

Vaccination can be an important component of a public health response to EBOV, especially for protection of healthcare workers and other individuals at high risk of infection during an ongoing EBOV outbreak in areas of poor infrastructure where biocontainment and contact tracing follow-up is often difficult and inadequate. Experience with control of other zoonoses including rabies^6^ and avian influenza^7^ also shows the potential impact of vaccination of intermediate host animals involved in transmission for control of zoonotic diseases such as EBOV.

Currently there are no licensed vaccines or therapeutic countermeasures against EBOV. However, the West African outbreak has focused attention on EBOV vaccine development. Over the past 15 years there has been remarkable progress in the development of EBOV vaccines using a variety of platforms including DNA, subunit, and several viral vector approaches (replicating and non-replicating), which have shown varying degrees of protective efficacy against EBOV in experimentally infected NHPs^8^. A number of these vaccine platforms have moved into clinical trials over the past year (www.ClinicalTrials.gov and www.pactr.org) with the aim of identifying efficacious vaccines for implementation to manage the current epidemic and to control future EHF outbreaks. To date all of these vaccines currently in human trials target the EBOV envelope glycoprotein, GP. Results from experimental animal studies using these vaccines indicate that antibody and cellular immunity can be important for protection against EBOV^8^. Interim results from an ongoing phase 3 cluster randomized trial indicate that at least one of these candidates, based on vesicular stomatitis virus, may be highly efficacious and safe^9^. It remains to be seen whether the other vaccine candidates currently in human clinical trials will prove as protective against EBOV, and which immunological mechanisms are involved.

Cytomegalovirus (CMV) is a ubiquitous β-herpesvirus that has gained considerable interest for development as a vaccine vector platform^10^,^11^,^12^,^13^. Recent studies have shown CMV vectors to provide a unique, T-cell-biased ‘effector’ (T_EM_) immune response^11^,^14^,^15^,^16^. T_EM_ cells localize predominantly to extralymphoid mucosal sites and are functionally primed for immediate effector function^17^, making them particularly useful to target pathogens that replicate and spread rapidly within the host. Rhesus macaque CMV (RhCMV) vectors expressing simian immunodeficiency virus (SIV) antigens under the control of constitutively expressed heterologous promoters provide a level of protection against systemic SIV that is suggestive of complete immunological clearance of SIV from vaccinated animals in this macaque model of AIDS^11^,^14^,^18^ – something that has not previously been observed with any other vaccine platform.

We recently showed the ability of a single dose of a murine CMV (MCMV) expressing a CD8^+^ T cell epitope from the nucleoprotein (NP) of EBOV (MCMV/ZEBOV-NP_CTL_) to provide protection against lethal mouse-adapted EBOV challenge in the mouse model, which corresponded to induction of durable, EBOV-specific CD8^+^ T cell immunity^15^,^16^. Although lowered levels of CMV viremia during secondary infection indicate that some control over CMV replication is afforded by pre-existing host immunity^19^, the capacity for CMV re-infection remains remarkably unaffected^20^,^21^ even for low inoculums of CMV (~10^2^ plaque forming units, pfu)^21^. This quality enables use of CMV-based vaccines in CMV-seropositive individuals, as well as the potential for serial re-use of CMV vectors encoding the same or different target antigen in the same individual^11^,^14^. CMVs are also highly species-specific, with the lack of CMV transmission between even closely related mammalian species^22^,^23^,^24^. The aim of the current study was to assess the protective efficacy of a RhCMV-based EBOV vaccine expressing full length EBOV GP (RhCMV/EBOV-GP) against a lethal EBOV challenge in CMV seropositive rhesus macaques, a translatable model for protection in humans^25^,^26^,^27^.

## Results

### Construction And Characterization Of RhCMV/EBOV-GP Vectors

**Figure 1:**
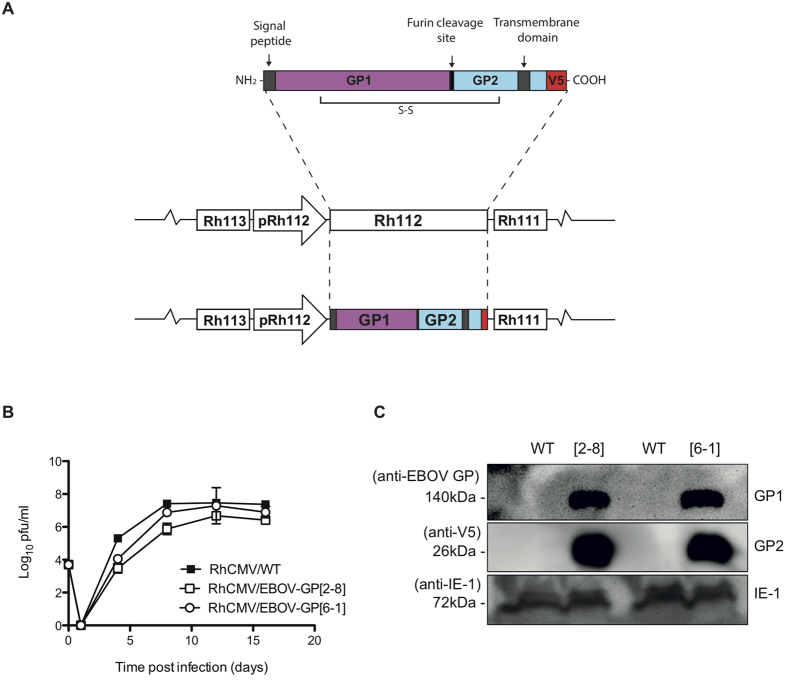
Construction and characterization of RhCMV vectors engineered to express EBOV GP (designated RhCMV/EBOV-GP). (**A**) Schematic representation of RhCMV/EBOV-GP. Codon-optimized full-length EBOV GP was inserted within the RhCMV genome (68.1) to replace the endogenous Rh112 (pp65b) ORF. This approach places GP under the control of the endogenous RhCMV Rh112 promoter. (**B**) Multi-step growth analysis of RhCMV/EBOV-GP. RFs were infected at a multiplicity of infection (MOI) of 0.01 with either RhCMV/WT, RhCMV/EBOV-GP[2–8] or RhCMV/EBOV-GP[6–1]. Supernatant was collected at days indicated post infection and titered using a TCID_50_ assay. The assay was performed in triplicate and standard deviation is shown. (**C**) Western analysis of RhCMV/EBOV-GP infected RF cell lysates showing stable expression of EBOV GP until at least passage 7. The EBOV GP was tagged at the carboxyl terminus with a V5 epitope to facilitate detection, and V5 epitope tag-specific monoclonal antibody (mAb) or a GP EBOV-specific mAb were used for detection. V5 activity was observed against 3 bands as predicted [26kDa GP2, 110kDa preGPer (full length endoplasmic reticulum form; not shown) and 160kDa preGP (full length Golgi form; not shown)]^55^. The GP-specific mAb was used to detect GP1 (140kDa) as the V5 tag is localized at the carboxyl-terminus of GP. Endogenous RhCMV IE-1 was used as an infection level control.

**Figure 2:**
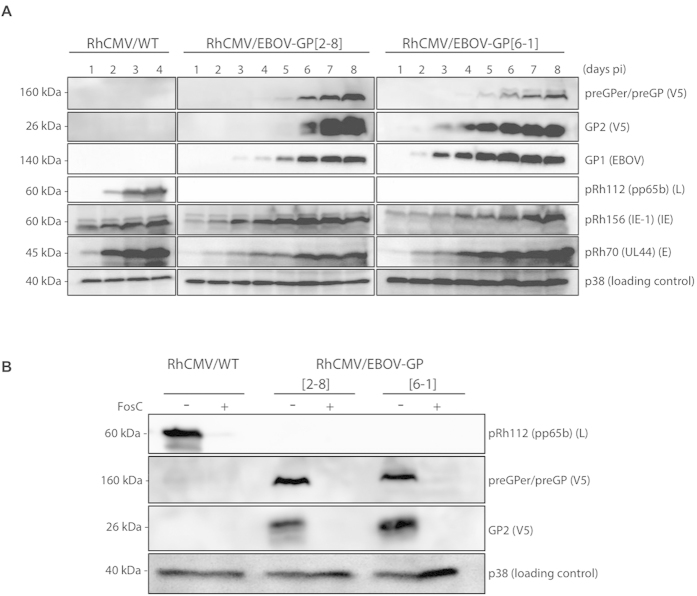
RhCMV/EBOV-GP expresses GP at late times of replication. (**A**) Western blot analysis of RhCMV/EBOV-GP in RFs showing EBOV GP expression at L times of replication. RFs were infected at a MOI of 0.03 with either RhCMV/WT, RhCMV/EBOV-GP[2–8] or RhCMV/EBOV-GP[6–1]. Cell lysates were collected at days indicated post infection and analyzed by western blot. Accumulation of GP was compared to accumulation of viral proteins known to be expressed with IE (IE-1), E (pRh70) and L (pRh112) kinetics. (**B**) Western blot analysis showing GP expression with L gene kinetics. RFs were infected at a MOI of 0.03 with either RhCMV/WT, RhCMV/EBOV-GP[2–8] or RhCMV/EBOV-GP[6–1] and treated with the CMV DNA polymerase inhibitor, foscarnet (200 μg/ml). Cell lysates were collected at indicated days post infection and analyzed for expression of indicated proteins. Absence of Rh112 and EBOV GP protein in the presence of foscarnet confirms expression of GP at L times of replication.

GP was chosen as the EBOV immunogen, as it has been used highly successfully with other vaccine platforms^8^. Vectors were constructed as described in Fig. 1A^11^,^14^. Previously published RhCMV-based vectors have generally used constitutively expressed heterologous promoters for target antigen expression^11^,^14^,^18^. In RhCMV/EBOV-GP the full length codon-optimized EBOV GP^28^ was instead placed under control of the endogenous RhCMV Rh112 (pp65b; pUL83b) promoter, which is one of the most highly expressed endogenous RhCMV promoters. This resulted in deletion of the RhCMV Rh112 open-reading frame (ORF), an ORF known to be dispensable for secondary RhCMV infection and virus persistence^29^. The ability of a recombinant RhCMV deleted for Rh112 to induce T cell responses against its heterologous expressed SIV target antigen similarly shows that Rh112 is not required for induction of T cell immunity in CMV-immune NHPs^29^. Fig. 1B shows *in vitro* replication kinetics of two independent RhCMV/EBOV-GP clones (2-8 and 6-1) in primary rhesus fibroblasts (RFs). Replication kinetics of RhCMV/EBOV-GP clones were delayed compared to parental WT virus (Fig. 1B), as we have observed previously for recombinant RhCMV expressing other heterologous antigens (unpublished results). GP expression was stable until at least passage 7 in RhCMV/EBOV-GP infected RFs (Fig. 1C). Similar to all herpesviruses, CMV genes differ in their time of expression during the virus replication cycle, being classified as immediate-early (IE), early (E) or late (L) genes. Expression kinetics revealed that GP was expressed in the late phase of RhCMV replication, consistent with its control by the L Rh112 promoter (Fig. 2A). The late expression of GP was confirmed by using the CMV DNA polymerase inhibitor, foscarnet, which blocks L gene expression (Fig. 2B).

### RhCMV/EBOV-GP Vectors Induce A Robust Anti-EBOV-GP IgG Response

**Figure 3:**
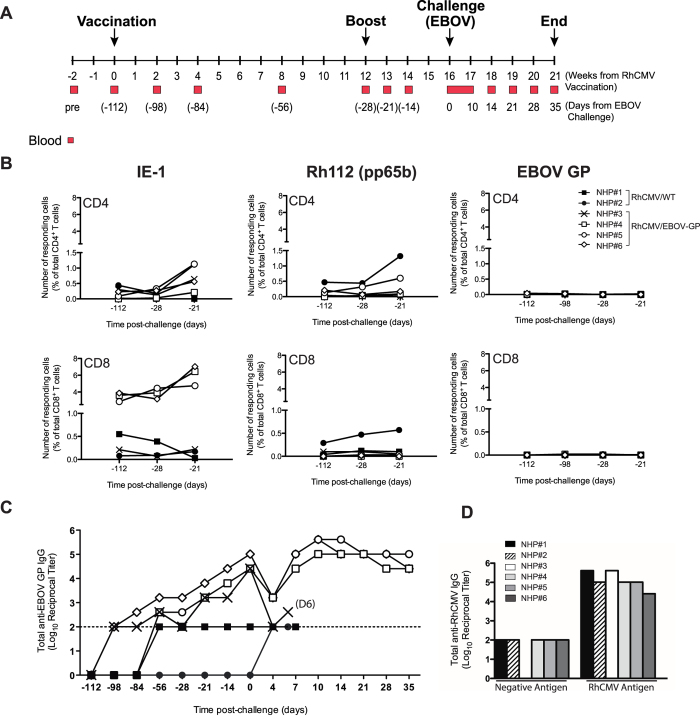
RhCMV/EBOV-GP induces high levels of EBOV GP-specific antibodies with absence of GP-directed T cell responses in NHPs. (**A**) Schematic showing timeline of ‘vaccine’ and ‘challenge’ phases and sampling schedule. (**B**) Time course of CD4^+^ and CD8^+^ T cell responses against IE-1, pRh112 (pp65b) and GP. T cells were analyzed by ICS following incubation with overlapping peptide pools in the presence of brefeldin (**A**). Levels of responding cells (TNFα and IFNγ double-positive) in individual NHPs are shown at indicated time points. T cell responses against endogenous RhCMV antigens (IE-1 and Rh112) were observed in all animals, while no responses against GP were detected at any time. (**C**) Time course of antibody responses against EBOV GP. Total EBOV GP-specific IgG levels were measured by ELISA at indicated time points. (**D**) RhCMV-specific antibody levels prior to vaccination. Presence of RhCMV-specific antibodies prior to vaccination (pre-bleed samples) indicates that all NHPs were RhCMV seropositive as a result of natural RhCMV infection at the time of initial vaccination.

To determine efficacy of this new vaccine vector against lethal EBOV challenge, a group of 4 NHPs (rhesus macaques) was inoculated with RhCMV/EBOV-GP (Fig. 3A). Two additional control animals received the parental 68-1 BAC-derived RhCMV^30^. All NHPs were already RhCMV seropositive as a consequence of natural RhCMV infection (Fig. 3D). At day -112, the 4 animals allocated to the vaccine arm were inoculated with 1 × 10^7^ pfu of RhCMV/EBOV-GP via the subcutaneous (s.c.) route. The 2 control animals received a comparable inoculation of 1 × 10^7^ pfu of parental 68-1 RhCMV. Animals were boosted in an identical fashion at day -28. NHPs were followed immunologically for T cell (Fig. 3B and Supp. Fig. 1) and EBOV-GP-specific IgG responses (Fig. 3C and Table 1). Previous studies using RhCMV vectors expressing SIV and human tuberculosis (TB) antigens under control of heterologous promoters, have shown immune responses against the target antigen to be shifted towards induction of cellular T_EM_-biased responses, with low or undetectable levels of antibodies^11^,^14^,^18^,^31^. We were therefore surprised to observe a reversal of this immunological phenotype, with RhCMV/EBOV-GP vaccination being associated with substantial levels of EBOV-GP-specific antibodies (Fig. 3C). Consistent with the capacity for serial use of CMV vectors, the RhCMV/EBOV-GP boost at day -28 resulted in an increase in GP-specific antibodies (Fig. 3C). Only background levels of CD4^+^ or CD8^+^ T cells were present against the GP antigen, even following the day -28 ‘boost’. Although variable, T cell responses against antigens encoded by endogenous RhCMV genes (IE1 and Rh112) were observed in all animals. This antibody-biased immune response directed against the heterologous target antigen (GP) is a phenotype not seen previously for any RhCMV-based vaccine^11^,^14^, or for other recombinant primate herpesvirus-based vectors^32^.

### RhCMV/EBOV-GP Vectors Protect Against Lethal EBOV challenge

**Figure 4:**
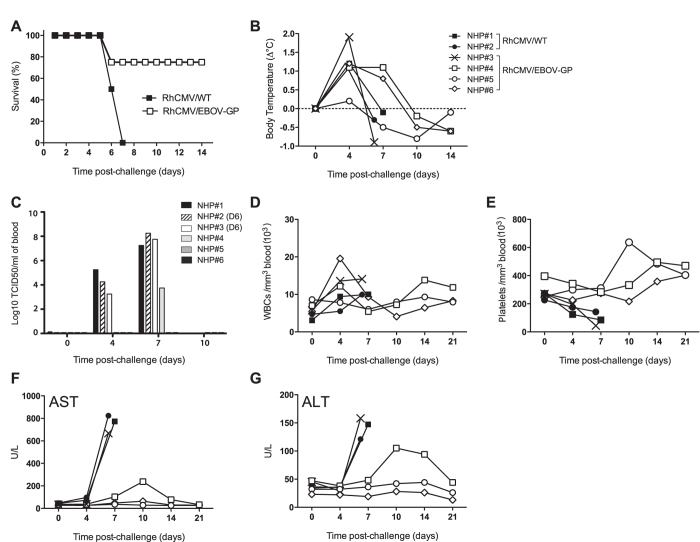
Clinical parameters in RhCMV/WT and RhCMV/EBOV-GP vaccinated animals. Changes in various clinical parameters were measured over the duration of the study. (**A**) Kaplan-Meier survival curves, (**B**) body temperature, (**C**) viremia, (**D**) white blood cell (WBC), and (**E**) platelet counts, and levels of (**F**) aspartate aminotransferase (AST) and (**G**) alanine aminotransferase (ALT).

To assess whether immunity induced by RhCMV/EBOV-GP protected animals from lethal EHF, the 6 NHPs were challenged with a lethal dose of EBOV at day 0. NHPs were monitored twice daily, and physical exams and blood draws were conducted on day 0, 4, 7, 10, 14, 21, 28, and 35 (Fig. 3A). Clinical findings are presented in Fig. 4A–G. Three of the 4 RhCMV/EBOV-GP vaccinated NHPs survived EBOV challenge (NHP#4, NHP#5 and NHP#6) indicating that vaccination had induced a protective immune response against EBOV. Two of the 3 protected animals were febrile (>1 °C above baseline) at day 4, but all animals returned to normal body temperature by day 10. Transient low-level viremia was observed in one animal (NHP#4) at a single time point (day 7), but viremia was undetectable in the remaining animals (NHP#5 and NHP#6) (Fig. 4C). One vaccinated animal (NHP#4) developed mild signs of disease, but survived. The 2 animals (NHP#1 and NHP#2), which received control vaccine were both febrile at day 4, and then rapidly developed EHF, reaching a predetermined clinical humane endpoint by days 6 and 7. A single animal from the vaccinated group (NHP#3) showed disease progression similar to controls, and was euthanized on day 6. Despite similar disease progression, the kinetics of viremia in NHP#3 were delayed, being 1- and 2-logs lower than control animals at day 4 (Fig. 4C), suggesting that RhCMV/EBOV-GP vaccination may have provided some low, partial level of protection in this animal. By day 6, EBOV levels in blood and tissues were comparable in NHP#3 and controls (data not shown). At this time, all 3 unprotected animals had severe thrombocytopenia and highly elevated levels of AST and ALT. Presence of macular cutaneous rash/petechiae over multiple areas in all of these animals was consistent with EHF. No rash was observed in any of the 3 protected RhCMV/EBOV-GP vaccinated animals.

### Protection Against Lethal EBOV Challenge Appears To Correlate With Anti-EBOV-GP IgG Levels

Given that the immunological phenotype for RhCMV-based vectors is normally heavily biased towards cellular T_EM_ memory with minimal antibody production^11^,^14^,^32^, the induction of a substantial anti-GP antibody response by RhCMV/EBOV-GP with an absence of detectable GP-specific T cells was unexpected. Although the present study was not powered for identification of the mechanism of protection for this vaccine, survival of the RhCMV/EBOV-GP immunized animals appeared to correlate with total EBOV GP-specific IgG level, as the single non-protected vaccinated animal (NHP#3) showed the lowest level of total GP-specific IgG (Fig. 3C). Recent cellular depletion studies in NHPs have confirmed that antibody rather than cellular GP-specific immune responses are of primary importance for recombinant vesicular stomatitis virus (rVSV)-EBOV-mediated protection^33^. The rapid decrease of anti-GP antibodies that we observed here in all the vaccinated animals at day 4 post challenge (Fig. 3C) has been seen before in EBOV immune animals following EBOV challenge^34^. Given the speed of decline (within 4 days of challenge) and the absence of leukocytopenia (Fig. 4D), this drop in antibody levels is likely caused by antibody-mediated consumption of EBOV. The decrease of GP-antibodies to below baseline levels in *only* the single vaccinated animal that died from EBOV disease is also persuasive of protection being afforded by a predominantly antibody-mediated mechanism. Neutralizing antibody titers were absent in vaccinated animals before challenge (day 0) and remained very low in protected animals after challenge (day 35) (Table 1), consistent with that observed for rVSV-EBOV vaccinated animals^35^. Additional studies powered to identify correlates of protection, as well as the use of depletion will be necessary to definitively determine the mechanism of RhCMV/EBOV-GP mediated protection.

## Discussion

Even though distinct immunological mechanisms appear to be at play, the present study shows that the protective immunity of CMV vectors initially suggested by mouse studies^15^,^16^ translates into protective efficacy using RhCMV-based vectors in the NHP EBOV challenge model. This study suggests a potential for development of CMV as prophylactic vaccine for ebolavirus in humans possibly using attenuated or replication-deficient CMVs. The relative contribution of antibodies compared to cellular immunity for protection against ebolavirus remains incompletely resolved^36^. In NHP experimental studies, rAd-based and rVSV-based ebolavirus vaccines appear to differ in their modes of protection – the former has been suggested to be primarily associated with cellular immunity^37^,^38^,^39^, whilst rVSV appears to be more antibody-mediated^33^. However, for both platforms total anti-GP antibody level correlates highly with survival from EBOV challenge in NHPs^40^, and total anti-GP antibody level has also been used as a primary immunological read-out for recent human clinical EBOV vaccine trials^9^,^41^,^42^. In the present study, the absence of substantial neutralization capacity of the EBOV antibodies induced by RhCMV/EBOV-GP implicates antibody effector mechanisms other than direct neutralization of virus infection in EBOV control, such as antibody-dependent cellular cytotoxicity (ADCC) or complement.

Vaccination with RhCMV/EBOV-GP induced substantial levels of EBOV-GP specific antibodies, but failed to induce the typical T_EM_ biased antigen specific T cell responses observed in other RhCMV-based vaccine vector studies^11^,^14^. Although one recent study in mice using a murine CMV-based vector has shown a similar phenotype^43^, this immunological shift towards antibodies has never been observed in macaques using RhCMV vectors, where responses are heavily T cell-biased with low antibodies induced against the heterologous target antigen^11^,^14^. The established capacity of rAd-expressed GP to induce GP-specific T cell responses^44^ indicates that the GP target protein does contain functional T cell epitopes. We hypothesize that the antibody-biased response phenotype seen in our study may result from expression of the GP target antigen being placed under control of the L Rh112 promoter. Expression at L times coincides with the expression of multiple CMV-encoded immunoevasins that downregulate MHC expression^45^. In our current model, the inability to present the heterologous target antigen to T cells via the canonical MHC pathway would shift immune responses away from cellular towards humoral immunity.

The high levels of GP antibodies induced by RhCMV/EBOV-GP and their ability to undergo IgG class switching indicates the presence of sufficient CD4^+^ T cell helper function. Given that CD4^+^ T cell responses against GP were below the level of detection in our study, this raises the possibility that CD4^+^ T cells directed against endogenous CMV proteins are functioning in *trans* to support the GP antibody response. This immune enhancement phenomenon has been observed in other systems, in particular during co-expression of the high-affinity Pan HLA-DR reactive epitope (PADRE) to supply CD4^+^ T cell help in *trans* to enhance immunogenicity against co-expressed target proteins^46^,^47^,^48^. The ability to modulate the adaptive immune response raises the possibility for rational design of vaccines that can direct the pathogen-specific immune response towards either antibody production with diminished T cell responses (by use of a promoter expressed at L times), or towards T cell responses with diminished antibody responses (by use of a promoter expressed at IE/E times). Using different kinetic classes of promoters to drive target antigen expression, either within distinct vaccines or within the same vaccine construct, would enable a balanced antibody and T cell response to be achieved. This raises the possibility of being able to recruit both arms of the immune response together against ebolavirus infection in a *single* CMV vaccine by differential promoter usage. The capacity to modulate the adaptive immune response may also have application to non-infectious disease, for example in preventing induction of meningoencephalitic T cell responses during antibody targeting of β-amyloid – a promising immunotherapeutic approach in preclinical and clinical trials as treatment for Alzheimer’s disease^49^.

Given its high-species specificity and ability to spread, even between CMV-seropositive individuals^20^,^50^,^51^, CMV may also be suited towards development as a ‘self-disseminating’ vaccine to target wildlife populations involved in EBOV zoonotic transmission (bats and great apes)^52^. Great apes (chimpanzees and western lowland gorillas) are one source of zoonotic ebolavirus transmission during human ebolavirus outbreaks, and are thought to represent a ‘dead-end’ host similar to humans^53^. Serological surveys and likely involvement of fruit bats in some human EBOV oubreaks similarly implicates bat species, most likely as a virus reservoir, in EBOV zoonotic transmission^53^,^54^. Use of a CMV-based ‘self-disseminating’ EBOV vaccine is one strategy that has been proposed to overcome the significant hurdles to providing protective EBOV-specific immunity in these inaccessible wildlife populations^15^,^16^,^52^. The present study used direct inoculation of animals for RhCMV/EBOV-GP vaccine administration, and immunity following animal-to-animal spread of the vector will now need to be investigated.

## Methods

### Animal Ethics and Biosafety Statement

All macaque work was performed in strict accordance with the recommendations described in the Guide for the Care and Use of Laboratory Animals of the National Institute of Health, the Office of Animal Welfare and the United States Department of Agriculture. Animal procedures were carried out under anesthesia by trained personnel under the supervision of veterinary staff and all efforts were made to promote the welfare and to minimize animal suffering in accordance with the “Weatherall report for the use of non-human primates” recommendations. Animals were housed in adjoining individual primate cages allowing social interactions, under controlled conditions of humidity, temperature and light (12-hour light/12-hour dark cycles). Food and water were available *ad libitum*. Animals were monitored at least twice daily (pre- and post-infection) and fed commercial monkey chow, treats and fruit twice daily by trained personnel. Environmental enrichment consisted of commercial toys. Humane endpoint criteria, specified and approved by the Institutional Animal Care and Use Committee (IACUC), were applied to determine when animals should be humanely euthanized. All infectious animal work was performed in the maximum containment laboratory at the Rocky Mountain Laboratories (RML), Division of Intramural Research (DIR), National Institute of Allergy and Infectious Diseases (NIAID), National Institutes of Health (NIH), Montana, USA applying standard operating protocols approved by the Institutional Biosafety Committee (IBC).

### Vaccine Vectors

We constructed RhCMV/EBOV-GP essentially as previously described^11^, by using E/T linear recombination to manipulate the parental RhCMV strain 68-1 genome cloned within a bacterial artificial chromosome (BAC)^30^. A codon-optimized version of GP (optZGP) from EBOV (Mayinga strain 76; Accession number AF086833) was used as the target antigen^28^. The optZGP open reading frame (ORF) was inserted within the RhCMV genome to replace the non-essential endogenous RhCMV Rh112 (pp65b) ORF (nucleotide positions 111,240 to 112,868 of RhCMV; see schematic Fig. 1A)^56^. This strategy places optZGP under control of the Rh112 promoter. The optZGP was epitope-tagged at the carboxy terminus with a V5 epitope. RhCMV/EBOV-GP BAC clones were analyzed *in vitro* as previously described (data not shown)^11^. Recombinant RhCMV/EBOV-GP viruses were reconstituted by transfection of BAC DNA into RFs^30^. Stability of optZGP expression in RhCMV/EBOV-GP vectors over at least 7 passages was confirmed by western analysis of infected cell lysates using monoclonal antibodies directed against EBOV GP1 and against the V5 epitope tag (Invitrogen) (Fig. 1C). Multi-step growth analysis of the RhCMV/EBOV-GP was performed as described^11^.

### Challenge Virus

EBOV strain Mayinga (passage 5) was propagated on Vero E6 cells (mycoplasma negative), titrated on these cells and stored in liquid nitrogen. Deep sequencing confirmed the “7U phenotype” for this EBOV strain.

### SDS-PAGE and western blotting

RFs infected at a multiplicity of infection (MOI) of 0.03 with either RhCMV/WT, RhCMV/EBOV-GP[2–8] or RhCMV/EBOV-GP[6–1] were solubilized in 1X Lamelli’s sample buffer, homogenized using QiaShredders (Qiagen), heated to 100 °C for 10 min, loaded onto 12% polyacrylamide gels and run in Tris-glycine buffer. Proteins were transferred to PVDF membranes (Bio-Rad) and blocked with 5% skimmed milk powder in Tris-buffered saline with 0.01% Tween-20 (TBST) overnight at 4 °C. Primary and secondary antibodies (HRP labeled; Bio-Rad) were applied at indicated dilutions: anti-V5 (1:1,500), anti-GP1 (1:10,000), anti-Rh112 (pp65b) (1:100), anti-Rh156 (IE-1) (1:500), anti-Rh70 (UL44) (1:2), anti-p38 (Santa Cruz Biotechnology, Inc.) (1:500), and secondary anti-mouse HRP (1:2,000) and anti-rabbit HRP (1:2,000). All antibodies were incubated for 1 hour at room temperature followed by three 5 minute washes with TBST. Membranes were developed using either ECL Western blotting substrate (Thermo Scientific) or SuperSignal West Femto substrate (Thermo Scientific), and imaged using a UVP ChemiDoc-It^2^ imaging system.

### Study design

Six adult male and female NHPs of Indian genetic background were used for the study. Animals were confirmed to be RhCMV seropositive by ELISA before initiation of the study (resulting from natural RhCMV infection) (Fig. 3D). Animals were filovirus-naïve and were also free of cercopithicine herpesvirus 1, simian T-lymphotropic virus type 1, D-type simian retrovirus and simian immunodeficiency virus. Animals were assigned to either RhCMV/EBOV-GP vaccine (n = 4) or parental wild-type RhCMV/WT control (n = 2) groups with an aim of achieving a relatively equal distribution based on sex and age. On day -112, the vaccine group received a single s.c. bolus of a mixture of two independent clones of the RhCMV/EBOV-GP construct (5 × 10^6^ pfu/construct). RhCMV/EBOV-GP and RhCMV/WT were administered subcutaneously as this has been the route used for inoculation of rhesus macaques in all earlier studies using RhCMV-based vectors (in this case, expressing SIV antigens^11^,^14^,^18^). The RhCMV/WT control group received a single 1  ×  10^7^ pfu s.c. inoculation of parental RhCMV/WT (clone 68-1)^30^. Animals were boosted with RhCMV/EBOV-GP clones or RhCMV/WT at week 12 (day -28). Blood samples were collected at times indicated over the pre-challenge 112 day period (vaccination phase) (Fig. 3A). Peripheral blood mononuclear cells (PBMCs) and plasma were prepared from blood by centrifugation on a histopaque gradient (Sigma) and assayed as detailed below. On day 0 (112 days post-vaccination), all animals were challenged with a lethal dose of 1,000 focus forming units of EBOV (strain Mayinga) by intramuscular administration at two anatomical locations (left and right caudal thigh). Animals were monitored twice daily for clinical signs of disease. Disease progression was assessed based on pre-established endpoints, and animals were humanely euthanized when clinical signs indicated onset of terminal disease. Blood samples were collected at times indicated (Fig. 3A) over the 35 day post-challenge period.

### Hematology and serum chemistry

A HemaVet^®^ 950FS laser-based hematology analyzer (Drew Scientific) was used to analyze the following blood parameters in 20 μl volumes of EDTA-treated blood: i) total white blood cell count, ii) lymphocyte, platelet, reticulocyte and red blood cell counts, iii) hemoglobin, iv) hematocrit values, and v) mean corpuscular volume and hemoglobin concentrations. Serum chemistry was analyzed using a Piccolo Xpress Chemistry Analyzer using Piccolo General Chemistry 13 Panel discs (Abaxis).

### Viral loads

Levels of infectious EBOV were measured by using standard virus titration^33^, followed by calculation of 50% tissue culture infectious dose (TCID_50_) using the method of Reed and Muench^57^. Tissues were homogenized prior to analysis.

### Intracellular cytokine staining analysis of T cells

Frequencies of CD4^+^ and CD8^+^ T cells directed against the EBOV (Mayinga) GP target antigen, as well as RhCMV IE-1 and Rh112 proteins were determined during the vaccine phase by intracellular cytokine staining (ICS) as previously described^33^. For stimulation, PBMC (1–2 × 10^6^ cells/well) were incubated *in vitro* with peptide pools (1 μg/ml final concentration) of overlapping peptides (11-mer with 5 amino acid overlap) representing each of the target ORFs. Incubation without antigen served as a background control. After 1 hour, brefeldin A (10 μg/ml) was added and cells were incubated for an additional 14 hours. Cells were surface stained using the following mAbs in indicated combinations: CD3, CD4 (eBioscience) and CD8β (Beckman Coulter). Cells were fixed and permeabilized according to manufacturer’s recommendations (BioLegend) prior to staining for intracellular staining using mAbs against Ki67 (BD) and IFNγ and TNFα. Polychromatic flow cytometric analysis was performed on a LSR II (BD Biosciences), and data was analyzed by using FlowJo software (version 10; Tree Star, Inc.). Response frequencies were determined by subtracting background and then averaging background subtracted responses.

### Enzyme-linked immunosorbent assay (ELISA)

Total IgG antibody responses to RhCMV/EBOV-GP were measured by ELISA using either EBOV GPΔTM or RhCMV/WT infected cell lysate as a source of antigen, as previously described^58^. The end-point dilution titer (using a 4-fold dilution series) is shown. Post-challenge plasma samples were inactivated by γ-irradiation (5Mrad) before removal from BSL-4 containment under standard RML operating procedures as approved by the RML Institutional Biosafety Committee (IBC). Samples were deemed positive for EBOV GP-specific IgG when the OD value was higher than the mean plus 3 standard deviations of negative (RhCMV WT) sera^59^.

### Neutralization assay

Neutralizing antibody titers were determined by performing focus reduction neutralization titration assays. Briefly, Vero E6 cells (mycoplasma negative) were seeded into 96-well plates to generate a confluent monolayer on the day of infection. Two-fold serum dilutions were prepared in triplicate in plain DMEM and 25 μl were incubated with 200 pfu EBOV (strain Mayinga) in a total volume of 50 μl. After 60 min at 37 °C the media was removed from cells, the serum-virus mixture was added and samples were incubated for 60 min at 37 °C. The mixture was then removed and 100 μl of 1.2% carboxymethyl cellulose in MEM (2% FBS) was added per well followed by incubation for 4 days at 37 °C. Cells were fixed in 10% neutral buffered formalin and removed from the maximum containment laboratory according to approved standard operating procedures (SOPs). Foci based either on GFP or staining using an anti-EBOV VP40 polyclonal rabbit serum and a secondary anti-rabbit FITC antibody (Sigma). Foci were counted and the neutralizing activity was determined as percent reduction of EBOV infection compared to control infected cells without plasma. Negative control: EBOV naïve rhesus macaque plasma. Positive control: NHP plasma of EBOV survivor.

### Statistical analysis

Kaplan-Meier survival curves were performed to visualize survival rates between groups in EBOV challenge studies. Analysis was performed using Prism GraphPad Software (Version 5.0d).

## Additional Information

**How to cite this article**: Marzi, A. *et al.* Cytomegalovirus-based vaccine expressing Ebola virus glycoprotein protects nonhuman primates from Ebola virus infection. *Sci. Rep.* **6**, 21674; doi: 10.1038/srep21674 (2016).

## Supplementary Material

Supplementary Information

## Figures and Tables

**Table 1:** RhCMV/EBOV-GP induces low levels of EBOV neutralizing antibodies.

Animal ID	Pre-challenge (D0)	Post-challenge (D35)
	**PRNT**_**50**_	
NHP#1	≤40	≤40
NHP#2	≤40	≤40
NHP#3	≤40	≤40
NHP#4	≤40	≤40
NHP#5	≤40	≤40
NHP#6	≤40	≤40
Negative control	≤40	
Positive control	160	

Neutralizing antibody titers were measured in vaccinated animals before challenge (day 0) and in protected animals after challenge (day 35). Shown is the plaque reduction neutralization test 50 (PRNT_50_) value, which is the highest plasma dilution that results in ≤50% of input plaque count (in absence of plasma).
