# Supplementary material for: Cytomegalovirus-based vaccine expressing Ebola virus glycoprotein protects nonhuman primates from Ebola virus infection
Source: Sci Rep. 2016 Feb 15;6:21674. doi: 10.1038/srep21674 (PMC4753684; doi:10.1038/srep21674)
Supplement: Supplementary Information [file srep21674-s1.pdf]

Cytomegalovirus-based vaccine expressing Ebola virus glycoprotein protects nonhuman primates from Ebola virus infection

Andrea Marzi[1,*], Aisling A. Murphy[2,*], Friederike Feldmann[3], Christopher J. Parkins[4], Elaine Haddock[1], Patrick W. Hanley[3], Matthew J. Emery[5], Flora Engelmann[6], Ilhem Messaoudi[6], Heinz Feldmann[1, †] & Michael A. Jarvis[2, †,§]

[1]Laboratory of Virology, Division of Intramural Research, and [3]Rocky Mountain Veterinary Branch, National Institute of Allergy and Infectious Diseases, National Institutes of Health, Hamilton, Montana, United States of America. [2]School of Biomedical and Healthcare Sciences, and [5]School of Biological Sciences, University of Plymouth, Devon, United Kingdom. [4]Vaccine and Gene Therapy Institute, Oregon Health & Sciences University, Portland, Oregon, United States of America. [6]School of Medicine, University of California, Riverside, California, United States of America.

[*,†]These authors contributed equally to this work.

[§]Correspondence should be addressed to M.A.J. (michael.jarvis@plymouth.ac.uk)

Supplementary Figure 1. Flow cytometry showing original dot-plot data presented from day -21 (1 week post-boost).

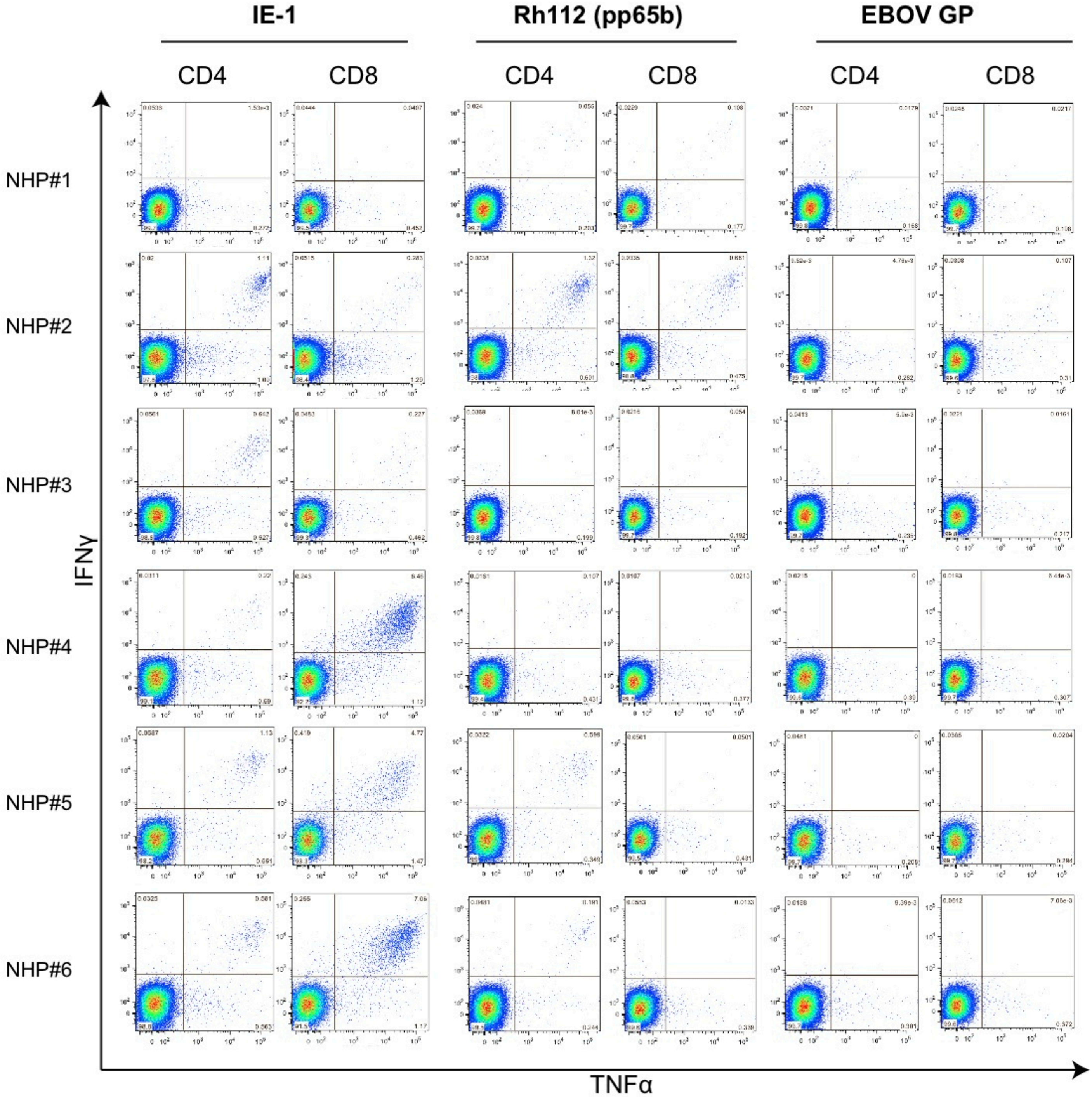

IE-1
Rh112 (pp65b)
EBOV GP
CD4
CD8
NHP#1
NHP#2
NHP#3
NHP#4
NHP#5
NHP#6
IFNγ
TNFα
